# Beta‐elemene inhibits breast cancer metastasis through blocking pyruvate kinase M2 dimerization and nuclear translocation

**DOI:** 10.1111/jcmm.14568

**Published:** 2019-07-25

**Authors:** Yanhong Pan, Wei Wang, Shuai Huang, Wenting Ni, Zhonghong Wei, Yuzhu Cao, Suyun Yu, Qi Jia, Yuanyuan Wu, Chuan Chai, Qian Zheng, Lei Zhang, Aiyun Wang, Zhiguang Sun, Shile Huang, Shijun Wang, Wenxing Chen, Yin Lu

**Affiliations:** ^1^ Jiangsu Key Laboratory for Pharmacology and Safety Evaluation of Chinese Materia Medica, School of Pharmacy Nanjing University of Chinese Medicine Nanjing China; ^2^ Department of Pharmacy, Anhui Provincial Hospital Anhui Medical University Hefei China; ^3^ Department of Biochemistry and Molecular Biology Louisiana State University Health Sciences Center Shreveport LA USA; ^4^ Shandong Co‐innovation Center of TCM Formula, College of Traditional Chinese Medicine Shandong University of Traditional Chinese Medicine Jinan China; ^5^ Jiangsu Collaborative Innovation Center of Traditional Chinese Medicine (TCM) Prevention and Treatment of Tumor Nanjing China

**Keywords:** aerobic glycolysis, beta‐elemene, breast cancer, metastasis, pyruvate kinase M2

## Abstract

Pyruvate kinase M2 (PKM2), playing a central role in regulating aerobic glycolysis, was considered as a promising target for cancer therapy. However, its role in cancer metastasis is rarely known. Here, we found a tight relationship between PKM2 and breast cancer metastasis, demonstrated by the findings that beta‐elemene (β‐elemene), an approved drug for complementary cancer therapy, exerted distinct anti‐metastatic activity dependent on PKM2. The results indicated that β‐elemene inhibited breast cancer cell migration, invasion in vitro as well as metastases in vivo. β‐Elemene further inhibited the process of aerobic glycolysis and decreased the utilization of glucose and the production of pyruvate and lactate through suppressing pyruvate kinase activity by modulating the transformation of dimeric and tetrameric forms of PKM2. Further analysis revealed that β‐elemene suppressed aerobic glycolysis by blocking PKM2 nuclear translocation and the expression of EGFR, GLUT1 and LDHA by influencing the expression of importin α5. Furthermore, the effect of β‐elemene on migration, invasion, PKM2 transformation, and nuclear translocation could be reversed in part by fructose‐1,6‐bisphosphate (FBP) and L‐cysteine. Taken together, tetrameric transformation and nuclear translocation of PKM2 are essential for cancer metastasis, and β‐elemene inhibited breast cancer metastasis via blocking aerobic glycolysis mediated by dimeric PKM2 transformation and nuclear translocation, being a promising anti‐metastatic agent from natural compounds.

## 1 INTRODUCTION

Cancer has become one of the leading causes for clinical death worldwide.^1^, ^2^ Particularly, metastasis accounts for over 90% cancer‐related death. Metastasis is a complicated multistage process including cancer cells detaching from primary site, intravasating into the blood or lymphatic vessel, surviving in circulation, extravasating the blood or lymphatic vessel and colonizing at a distant organ.^3^, ^4^ Although hypoxia is an important inductive factor for carcinogenesis and metastasis,^5^ glycolysis was considered a prior process for cancer development and progression in the presence of oxygen, namely aerobic glycolysis.^6^

Since the theory of aerobic glycolysis was created by Otto Warburg in 1930s and emphasized again in recent decades, cancer has been regarded as a metabolic disease. Cancer cells need fast and sufficient production of ATP to support proliferation and survival during the cancer initiation and progression. Oxidative phosphorylation produces ATP more efficiently, whereas aerobic glycolysis produces ATP more rapidly. Cancer cells attain ATP preferably by the rapid aerobic glycolysis rather than the efficient oxidation even if the oxygen exists. Also, lots of critical precursors that promote cancer progression are produced by aerobic glycolysis but not oxidative phosphorylation.^6^

There are three important rate‐limiting enzymes including pyruvate kinase (PK), phosphofructokinase (PFK) and hexokinase (HK) to control the aerobic glycolysis. Among them, PK regulates the final step of glycolysis, being considered as a central kinase in reprogramming the cellular metabolism.^7^ The isoforms of PK include PKR, PKL, PKM1 and PKM2, the former two are generally expressed in liver and erythrocytes, while the latter two distributed ubiquitously in all types of cells and tissues. Specially, PKM2 has attracted great concern as a target kinase for cancer therapy. Studies have demonstrated that PKM2 possesses multifaceted metabolic and non‐metabolic functions to regulate cancer progression.^8^ Firstly, PKM2 expression differs between normal cells and cancer cells, which is regulated by many signalling pathways involving hypoxia‐inducible factor 1‐alpha (HIF‐1α), nuclear factor kappa‐light‐chain enhancer of activated B cells (NF‐κB) and specificity protein 1 (SP1), DNA methylation and PKM pre‐mRNA precursor, and controlled at transcriptional, post‐transcriptional and epigenetic levels.^9^ Secondly, PKM2 can be mutually converted in conformation between dimer and tetramer, having low and high affinity to its substrate phosphoenolpyruvate (PEP), respectively.^9^ Oncogenic tyrosine kinases including fibroblast growth factor receptor (FGFR), BCR‐ABL and Janus kinase 2 (JAK2) can regulate the PKM2 activity through modulating the dimer‐tetramer conversion.

**Figure 1:**
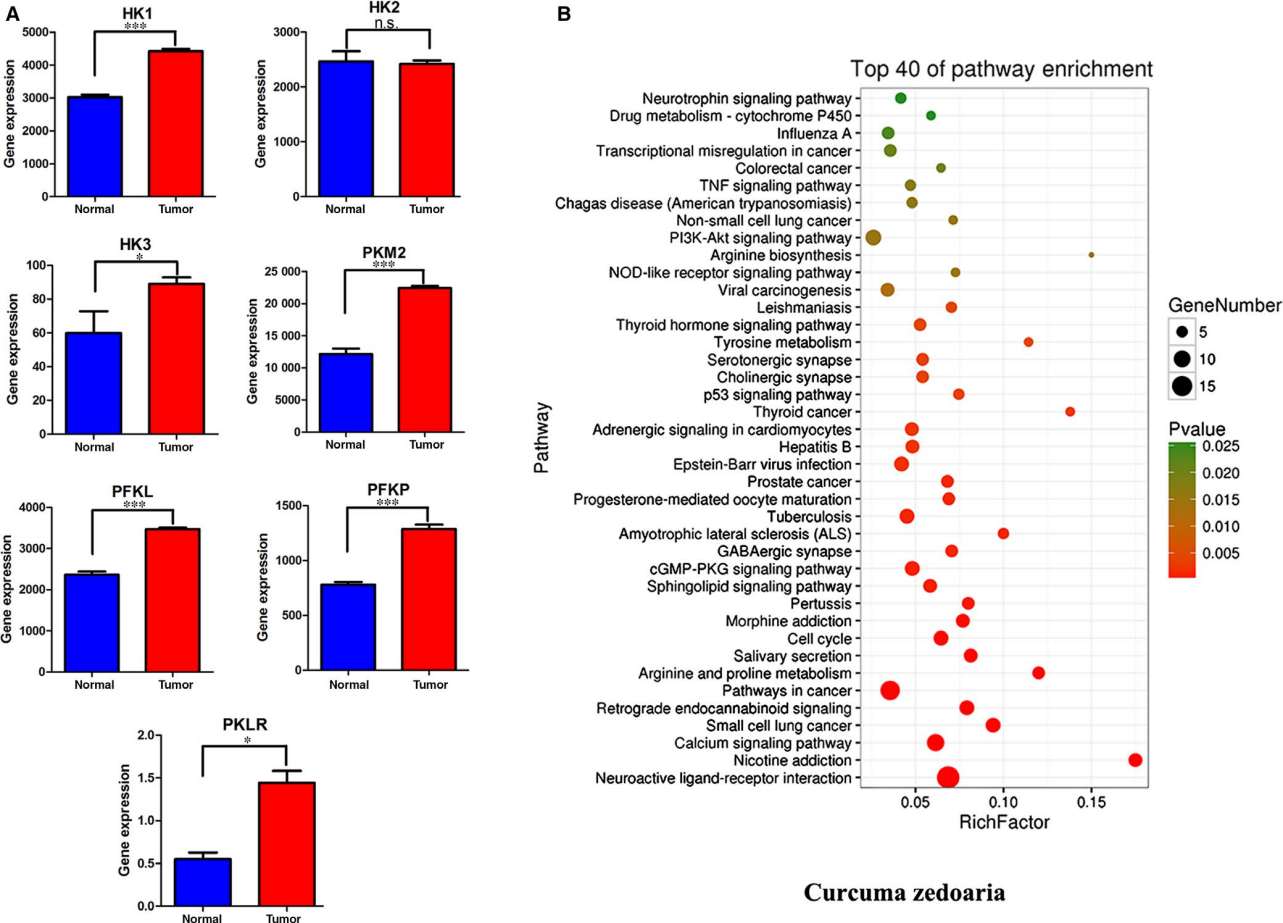
Gene expression of HK, PFK and PK in breast cancer patients as well as enrichment analysis of putative targets of *Curcuma Zedoary*. A, The gene expression of HK, PFK and PK in breast cancer patients. It was based on BRCA.rnaseqv2 data file in the TCGA database. After genetic screening, samples of repetitive types of the same patient were removed according to hybridization REF which differentiates sample types. 112 cases of normal tissue samples and 1100 cases of tumour tissues were selected. The data were analysed with one side *t* test by GraphPad Prism Software. Normal tissue versus tumour tissue, *P<.05, ***P < .001. In this analysis, cancer stage and gene mutation were not considered. B, The enrichment analysis of putative targets for *Curcuma Zedoary*

Breast cancer is the most commonly diagnosed carcinoma with high mortality all over the world 1, 2, 10 and is also one type of the carcinoma with high metastatic properties. Through analysing the mRNAs of the tissues from the patients with breast cancer in the TCGA database, the genes related to glycolysis including HK, PFK and PK are expressed significantly higher in the cancer tissues than normal tissues (Figure 1A), demonstrating that the progression and metastasis of breast cancer are related to glycolysis.

Beta‐elemene (β‐elemene), a major non‐cytotoxic anticancer ingredient isolated from *Curcuma Zedoary*, can induce cancer cell apoptosis, cell cycle arrest, and enhance the sensitivity of radiotherapy and chemotherapy without myelosuppression and obvious damages to liver and kidney.^11^, ^12^, ^13^, ^14^ β‐Elemene has already been approved by China food and drug administration (CFDA) to against cancer in clinic, in combination with chemotherapeutic drugs. Furthermore, β‐elemene not only indicated significant inhibition on melanoma metastasis in vitro and in vivo,^15^ but also enhanced the chemotherapeutic effect on the patients with metastasis based on the meta‐analysis of clinical data.^16^ However, the molecular mechanism of β‐elemene inhibition of metastasis is unknown. *Curcuma Zedoary* is a traditional Chinese medicine used frequently in clinic and contains many ingredients with multiple activities. To unveil the relationships between the ingredients and targets, we conducted the enrichment analysis of putative targets for *Curcuma Zedoary* for breast cancer. By combining oral bioavailability (OB) screening data in the TCMSP website, we identified 91 active ingredients (compounds) in *Curcuma Zedoary*. An online database (QuickGO) and DAVID bioinformatic database for annotation, visualization and integrated discovery 17, 18 were utilized for gene ontology (GO) analysis of the putative targets of *Curcuma Zedoary*, showing that the putative targets were enriched in the pathways related to cellular metabolism, such as calcium signalling pathway, p53 pathway 19 and amino acid metabolism,^20^ which are tightly associated with cancer metastasis (Figure 1B).^20^, ^21^

In this study, we focused on the relationship between breast cancer metastasis and metabolism and found that breast cancer metastasis was regulated by PKM2 transformation and nuclear translocation; β‐elemene could inhibit breast cancer metastasis by down‐regulating aerobic glycolysis via blocking transformation and nuclear translocation of PKM2.

## 2 MATERIALS AND METHODS

### 2.1 Chemicals and bioreagents

β‐Elemene (purity 98%) was from Isobionics (Geleen, the Netherlands). Elemene injection (the content of β‐elemene >95%) was obtained from Jingang Pharmaceuticals. DMEM, RPMI 1640, foetal bovine serum (FBS) were obtained from Gibco as well as Trypsin‐EDTA (0.05%) and penicillin/streptomycin. D‐(+)‐glucose, sodium pyruvate and L‐glutamine were provided by Sigma‐Aldrich. Other standard chemical compounds were obtained from Yuanye Biotechnology Co., Ltd. ATP determination kit was from Molecular Probes of Invitrogen.

### 2.2 Cell proliferation assay

Human breast cancer cells (MDA‐MB‐231 and MCF‐7) were from American Type Culture Collection and maintained as suggested by the supplier. Both cells were tested for mycoplasma contamination. The evenly dispersed cancer cells by a density of 10^4^ cells/well were piped into a 96‐well plate. After overnight incubation, the cells were mixed with the corresponding concentrations of β‐elemene (final concentration in the medium: 0‐320 μmol/L) for 48 hours. Then, 20 μL of the prepared MTS reagent was added into each well for chromogenic reaction. And the optical density indicating the cell viability was measured at 570 nm by a Biotek microplate reader (Biotek Corporation).

### 2.3 Wound healing assay

As described,^22^ wound healing assay was performed. Briefly, MDA‐MB‐231 cells (1.5 × 10^6^ cells/well) and MCF‐7 cells (1 × 10^6^ cells/well) were seeded into 6‐well plates and grown into 100% confluent monolayer culture. Mitomycin C (10 μg/mL) was added to inhibit cell proliferation. After 1‐hour incubation, the confluent monolayer cells were wounded by a sterile pipette tip, followed by replacing with the basal medium containing different concentrations of β‐elemene or dimethyl sulfoxide (DMSO). The cell migration images were photographed under a ZEISS microscope at 0, 24 and 48 hours following scraping at 3‐4 different locations. The wound closure was measured by ImageJ 1.5.

### 2.4 Transwell migration and invasion assay

A 24‐well transwell system (Corning, NY, USA) was chosen for evaluation of cell migration and invasion. The transwell chamber system was used directly for cell migration, while the one coated with 1 mg/mL Matrigel (Corning, NY, USA) for cell invasion. MDA‐MB‐231 and MCF‐7 cells (4 × 10^5^ cells per transwell), after treated with the basal medium for 24 hours, were added into the upper chamber with serum‐free medium containing β‐elemene (5, 10, 20 and 40 μmol/L) or DMSO as a control, and 800 μL of the medium with 30% FBS was piped into the lower chamber. After incubation in a 37°C incubator with 5% CO_2_, the medium in the upper chamber was removed and the non‐migrating or non‐invading cells were cleaned by a cotton swab. The remaining cells were fixed, stained and washed by 4% paraformaldehyde, 1% crystal violet solution and phosphate‐buffered saline (PBS), respectively, in turn. The number of invaded and migrated cells was counted at 200× magnification by six fields of view for average.

### 2.5 Measurements of OCR and ECAR

The Seahorse XF Glycolysis Stress Test Kit and the Seahorse XF^e^24 Extracellular Flux Analyzer (Seahorse Biosciences) were used to determine the glycolytic function in cells. The Seahorse XF Cell Mito Stress Test Kit (Seahorse Biosciences) was used to determine the cellular mitochondrial capacity. Cells seeded into a 24‐well plate (1 × 10^4^ cells/well) for overnight incubation were treated with fresh medium containing various concentrations of β‐elemene for 24 hours before the test operation. For Seahorse assay, the cells were washed with Seahorse assay medium. Next, interval injections of 10 μmol/L oligomycin, 2.5 μmol/L FCCP and 5 μmol/L rotenone/antimycin A were for determining the oxygen consumption rates (OCR). Then, 100 mmol/L glucose, 10 μmol/L oligomycin and 500 mmol/L 2‐deoxy‐glucose (2‐DG) were in turn added to determine the extracellular acidification rates (ECAR). The value of OCR and ECAR was calculated by normalization to the cell number.

### 2.6 Analysis of metabolites by HPLC‐MS

Except glucose, pyruvate and lactate were measured using specified reagent kits (Nanjing Jiancheng Biotech Co. Ltd) and HPLC‐MS for their extracellular and intracellular content; other metabolites were directly determined by HPLC‐MS. Cell and tissue lysates were extracted in the extraction buffer (containing methanol and water (1:1, v/v), 5 mmol/L ammonium acetate) in the cold room for 15 minutes, followed by scraping and centrifuging at 17 000 *g* for 10 minutes. Metabolic flux analysis was performed by liquid chromatography (Prominence LC‐20A, Shimadzu, JPN) with a tandem quadrupole mass spectrometry (QTRAP®5500, AB Sciex, MA, USA). Samples were injected into a 4.6 mm × 150 mm StableBond column (ZORBAX SB‐AQ 5 µm; Agilent). The chromatography was run started with 98% solution B (0.1% formic acid in H_2_O) and 2% solution A (0.1% formic acid in methanol), followed by 4 minutes going down to 80% solution B, 4.5 minutes coming back to 98% solution B, and then holding up until stopped. Data were collected and analysed by Analyst Software (AB Sciex).^23^

### 2.7 Spontaneous xenograft metastatic studies in mice

According to the protocol described previously,^24^ MDA‐MB‐231 or MCF‐7 cells (3 × 10^6^ cells/each injection) were subcutaneously injected into the mammary fat pad near the fourth nipple of BALB/c nude female mice (6‐8 weeks old, obtained from Nanjing Biomedical Research Institute of Nanjing University). The mice were randomly divided into two groups (8 mice/group). Furthermore, additional 4 mice with no injection of cancer cells and no treatment with β‐elemene but under the same environment were classified as the normal group. Then, the mice of the model and normal groups were intraperitoneally administrated with 5% glucose injection, while another group with β‐elemene (50 mg/kg) once a day. At 21‐day post‐treatment, all mice were sacrificed, the organs (liver and lung) were collected for corresponding assays. The animal study was approved by the Animal Ethics Committee of Nanjing University of Chinese Medicine. And the procedures were according to the guidelines for the Administration of Affairs Concerning Experimental Animals approved by the State Council of People's Republic of China.

### 2.8 Western blot analysis

Western blot was executed and analysed according to the reference.^25^ And the primary antibodies were used PKM2, p‐PKM2, GLUT1, MCT1, MCT4, EGFR (Abcam, Cambridge, UK), LDHA (Cell Signaling Technology), PIN1, importin α5, β‐actin and PKM (AB clonal).

### 2.9 Immunofluorescence staining

The glass coverslips seeded with cancer cells were plated in 6‐well plates for 24‐hour treatment of β‐elemene. Then, the coverslips were covered, respectively, by 4% paraformaldehyde for fixation, by 0.2% Triton X‐100 for permeabilization and by 1% BSA for blocking non‐specific antibody. And followed with incubation with corresponding primary antibody and secondary antibody, the coverslips were dyed in Hoechst in final. The analysis data were attained by fluorescent microscopy.

### 2.10 Immunohistochemistry

The lung and liver tissues from mice behind fixation in formalin and embedding in paraffin were cut into 5‐μm sections for IHC staining. Then, the sections were in turn retrieved for the antigen using citrate buffer (0.01 mL, pH 6.0), washed with PBS and incubated with endogenous peroxidase blockers for 10 minutes and with antibodies at 4°C overnight. Post‐incubation with reaction enhancer before horseradish peroxidase‐labelled antimouse IgG antibody for 20 minutes at room temperature; the slides were prepared with DAB and analysed by Mantra Workstation equipped with OLYMPUS BX43 (200×) (PerkinElmer).

### 2.11 Statistical analysis

Each experiment was executed in triplicate. The quantitated results were determined by one‐way ANOVA followed with one side *t* test by GraphPad Prism software. *P* < .05 represents a statistical significance.

## 3 RESULTS

### 3.1 β‐Elemene inhibited breast cancer cell migration and invasion

Although β‐elemene inhibition of proliferation and induction of apoptosis in many cancer cells were demonstrated, its anti‐metastatic activity and mechanism are rarely known. Here, we observed that β‐elemene had no inhibition on viability of MDA‐MB‐231 and MCF‐7 cells under 40 μmol/L (Figure S1A). So, the concentrations under 40 μmol/L were chosen for the subsequent experiments.

**Figure 2:**
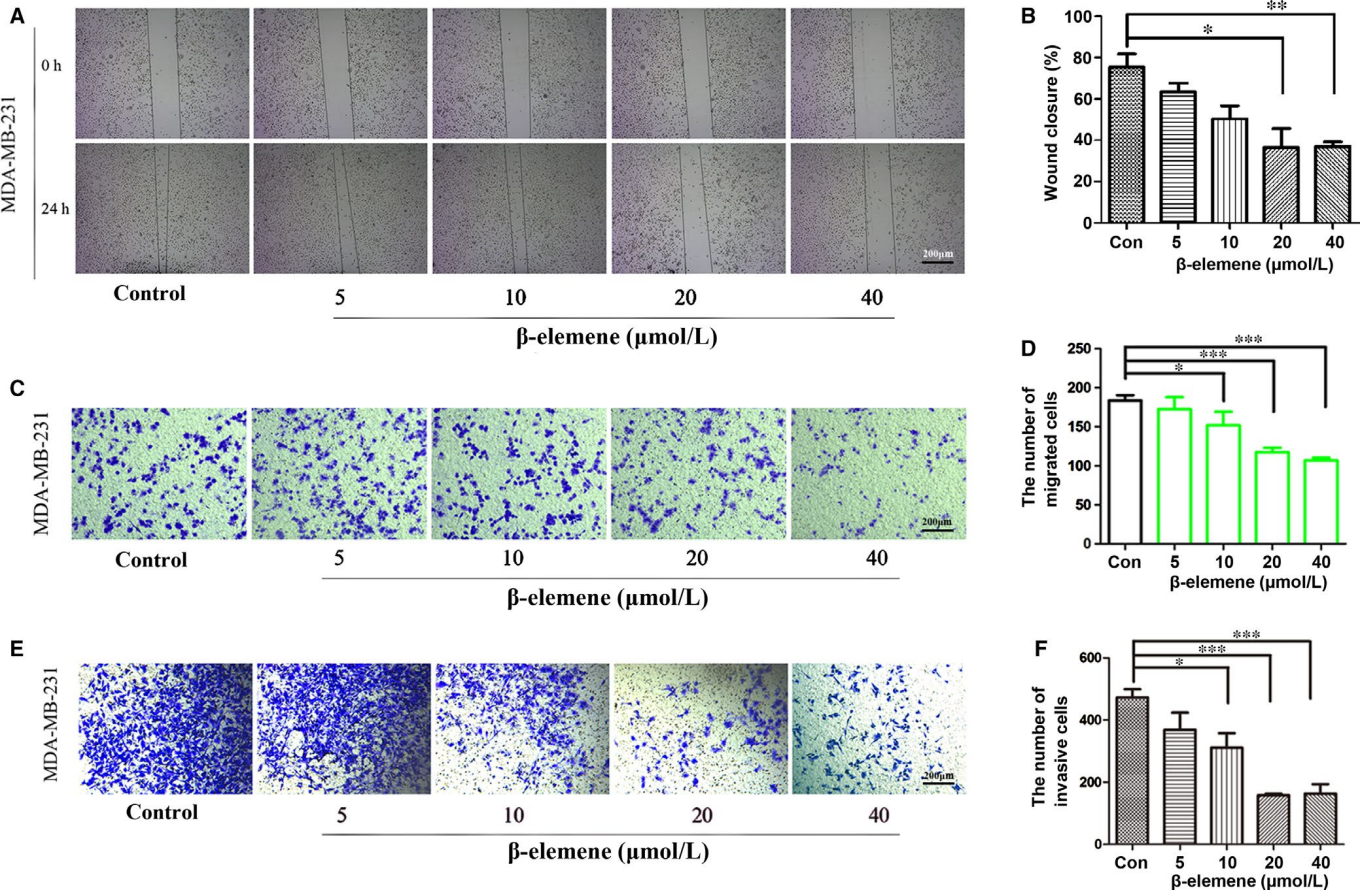
β‐Elemene inhibited breast cancer cell migration and invasion. A and B, Representative images (40×) of wound healing assay from different treated groups and quantitative analysis. C, D, Representative photographs (200×) of transwell migration assay and quantitative analysis. E, F, Representative photographs (200×) of transwell invasion assay and quantitative analysis. MDA‐MB‐231 cells were used in these experiments, and the quantitated data were for one‐way ANOVA with one side *t* test, versus control, *P < .05, **P < .01, ***P < .001

In the wound healing assay, MDA‐MB‐231 cells migrated faster than MCF‐7 (Figure 2A, Figure S1B). A 24‐h treatment with 20 and 40 μmol/L β‐elemene significantly blocked the migration of MDA‐MB‐231 cells (Figure 2A,B), and the similar results were repeated in MCF‐7 cells (Figure S1B,C). In the Matrigel‐Transwell assay, both MDA‐MB‐231 and MCF‐7 cells could successfully penetrate the Matrigel‐coated chambers. However, β‐elemene treatment (≥10 μmol/L) led to fewer cells migrating and invading the membrane matrix, compared with the control (DMSO) (Figure 2C‐F, Figure S1D‐F). Totally, the results indicated that β‐elemene was able to inhibit breast cancer cell migration and invasion.

### 3.2 β‐Elemene inhibited breast cancer metastasis in lung and liver in mice

**Figure 3:**
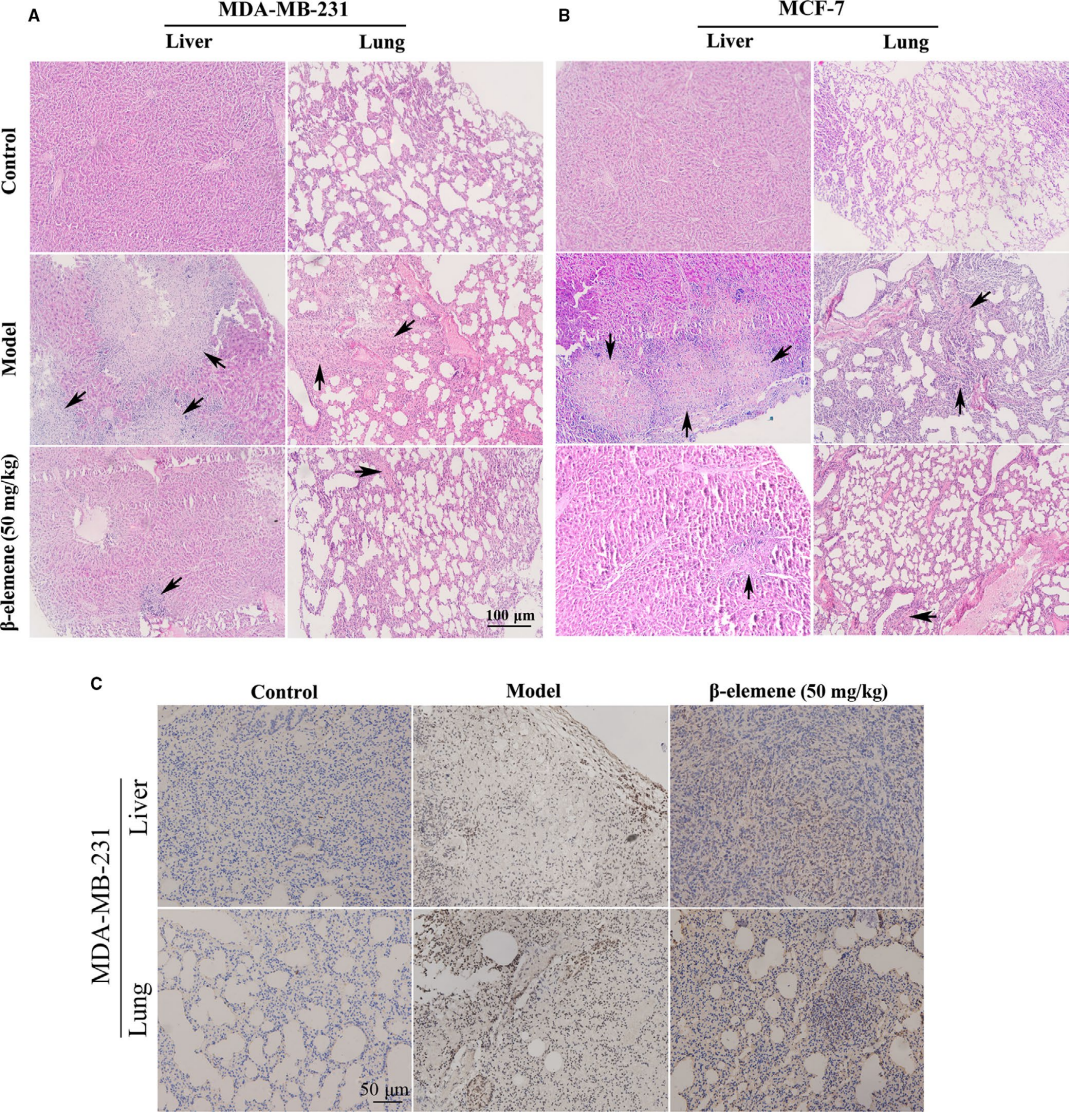
β‐Elemene inhibited metastases in lung and liver of high and low metastatic breast cancer models. A and B, H&E staining of the lung and liver tissues of mice xenografted with MDA‐MB‐231 (A) or MCF‐7 (B) cells (100×). Arrows indicate the metastatic foci. C, The immunochemistry staining for ki‐67 in metastatic organs (100×) from MDA‐MB‐231 transplanted mice

To further investigate whether β‐elemene inhibits the metastasis of breast cancer in vivo, MDA‐MB‐231 and MCF‐7 orthotopic xenograft mouse models were used. Mice were treated once a day for 21 days by intraperitoneal injection with β‐elemene (50 mg/kg body weight). The results indicated that the metastatic colonies widely distributed in whole lung and liver of the control mice, and calcification appeared in the liver, were effectively attenuated by 50 mg/kg β‐elemene (Figure 3A,B). Accurately, the areas of metastasis and calcification were obviously decreased. Furthermore, the metastatic foci in liver and lung indicated by ki‐67 antibody staining were decreased in 50 mg/kg β‐elemene treatment group (Figure 3C). Collectively, β‐elemene could inhibit breast cancer metastasis in lung and liver in vivo.

### 3.3 β‐Elemene inhibited aerobic glycolysis of breast cancer cells

**Figure 4:**
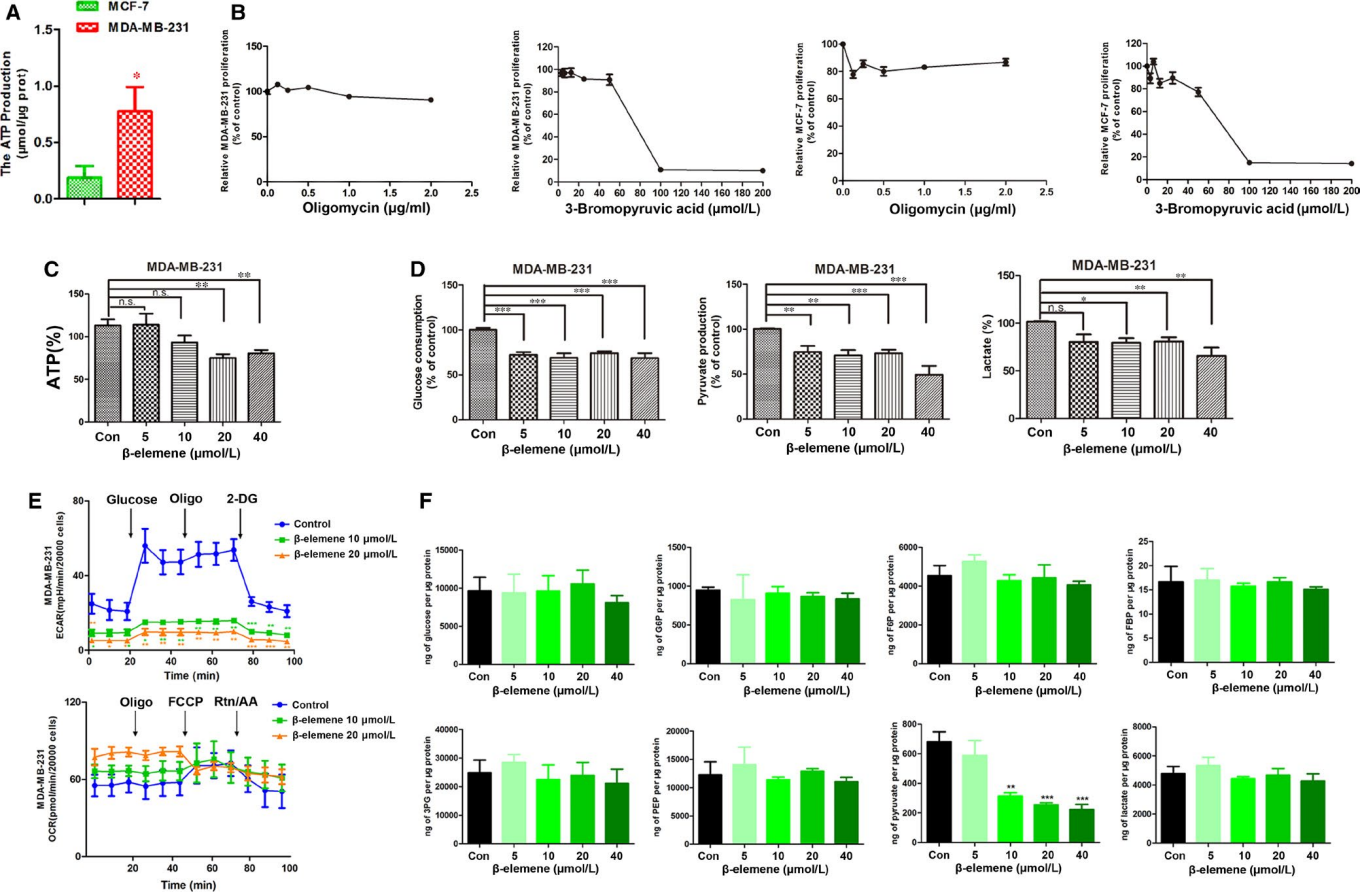
β‐Elemene inhibited aerobic glycolysis of breast cancer cells. A, The content of ATP in MDA‐MB‐231 and MCF‐7 cells. B, Cells were seeded in 96‐well plates and treated with 3‐bromopyruvic acid (0‐200 μmol/L) or oligomycin (0‐2.0 μg/mL) for 24 h with 6‐replicates of each treatment. 3‐Bromopyruvic acid, an inhibitor of glycolysis; oligomycin, an inhibitor of TCA. Cell proliferation was measured by MTS assay. C and D, The production of ATP (C), pyruvate and lactate and the consumption of glucose of different treated groups (D) in MDA‐MB‐231 cells. E, ECAR and OCR were determined in MDA‐MB‐231 cells treated with β‐elemene. F, The content of intracellular glucose, G6P, F6P, FBP, PEP, 3PG, pyruvate and lactate of different treated groups measured by HPLC‐MS. The quantitated data were for one‐way ANOVA with one side *t* test, versus control, *P < .05, **P < .01, ***P < .001

Studies have confirmed that metastasis is tightly associated with tumour metabolism.^26^, ^27^ Here, based on the results of enrichment analysis of putative targets for *Curcuma Zedoary*, we focused on whether the anti‐metastatic activity of β‐elemene is related to aerobic glycolysis. The results showed that low metastatic MCF‐7 cells produced less ATP than high metastatic MDA‐MB‐231 cells (Figure 4A). Both MDA‐MB‐231 and MCF‐7 cells are glycolytic phenotype, as the proliferation of the two cell lines was remarkably inhibited by 3‐bromopyruvic acid (a glycolytic inhibitor), while even 2 μg/mL oligomycin (a mitochondrial oxidative phosphorylation inhibitor) did not influence their proliferation (Figure 4B). This was also supported by the control data in Figure 4E and Figure S2C. It was indicated that MDA‐MB‐231 was a complete glycolytic phenotype because ECAR had a significant change following addition of glucose or 2‐DG but OCR showed no change when added oligo or FCCP; MCF‐7 was an incomplete glycolytic phenotype as the curve of ECAR and OCR was standard like most of cancer cells. This also drew a conclusion that metastasis was highly related to the aerobic glycolysis.

Compared with the control, β‐elemene decreased the production of ATP (Figure 4C, Figure S2A), the consumption of glucose, the production of pyruvate and lactate in MDA‐MB‐231 (Figure 4D) and MCF‐7 cells (Figure S2B). However, the change of intracellular and extracellular glucose and lactate levels was different. To further uncover how β‐elemene affected glucose metabolism, we determined the OCR and ECAR values using the XF^e^24 Extracellular Flux analyser. The results indicated that 10 and 20 μmol/L β‐elemene caused a decrease in ECAR in both MDA‐MB‐231 and MCF‐7 cells (Figure 4E, Figure S2C). However, the effect of β‐elemene on the OCR differed in the two cell lines, as β‐elemene reduced the OCR of MCF‐7 but not MDA‐MB‐231 cells (Figure 4E, Figure S2C). HPLC‐MS analysis of the metabolites of glucose revealed that the content of pyruvate was significantly decreased in both two cell lines, but the levels of glucose‐6‐phosphate (G6P), fructose‐1,6‐bisphosphate (FBP) and lactate were only reduced in MCF‐7 cells (Figure 4F, Figure S2D). Above results indicated that β‐elemene inhibited the aerobic glycolysis of breast cancer cells.

### 3.4 β‐Elemene blocked PKM2 transformation and nuclear translocation

**Figure 5:**
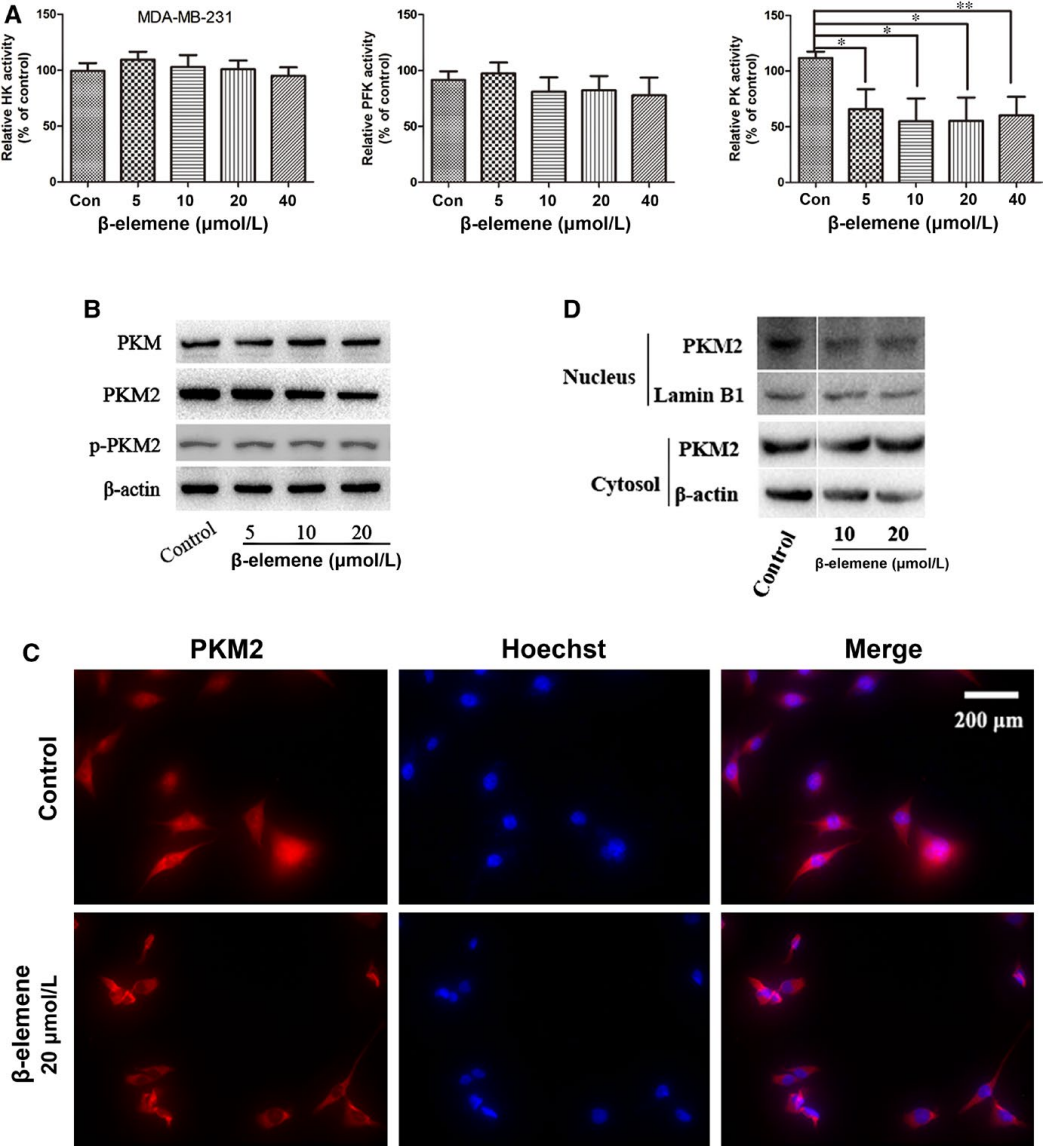
β‐Elemene inhibited PKM2 transformation and nuclear translocation. A, The activity of HK, PFK and PK in MDA‐MB‐231 cells with indicated treatments. B, The Western blotting for PKM, total PKM2 and p‐PKM2 in MDA‐MB‐231 cells with indicated treatments. C, The nuclear translocation of PKM2 after 24‐h β‐elemene treatment. D, The Western blot assay for the cytoplasmic and nuclear expression of PKM2. The quantitated data were for one‐way ANOVA with one side *t* test, versus control, * *P* < .05, **P < .01

To further reveal the mechanism by which β‐elemene inhibited glycolysis of breast cancer cells to regulate metastasis, we investigated the effects of β‐elemene on three key enzymes HK, PFK and PK which ensures that glycolytic intermediates are fully utilized and transported into the appropriate metabolic pathway to balance energetic and anabolic demands. The data showed that β‐elemene inhibited PK activity, which catalyses the last step of glycolysis, but had no effects on the activities of HK and PFK (Figure 5A, Figure S3A). Furthermore, β‐elemene did not affect the protein levels of PKM, total PKM2 and phosphorylation of PKM2 in both cells (Figure 5B, Figure S3B). PKM2 is normally present in the cytoplasm by a form of homotetramer and functions as a metabolic kinase with high catalytic activity.^8^ In addition, a dimeric PKM2 form has been recently confirmed in cell nucleus, while it works as a protein kinase stimulating transcription to regulate the growth, survival and metastasis of tumour cells.^8^, ^28^ In our study, we discovered that β‐elemene suppressed PKM2 translocating into the nucleus. This was supported by the data that in the control, PKM2 expressed in the whole cell by the immunofluorescent staining, but just in the cytoplasm under the β‐elemene treatment (Figure 5C, Figure S3C), and PKM2 expression by the Western blot assay was obviously inhibited in the nucleus, but increased in the cytoplasm (Figure 5D, Figure S3D). Thus, it was confirmed that β‐elemene reduced the ratio of tetrameric/dimeric PKM2 leading to inhibit the tetramer PKM2 transformation to dimer. Meanwhile, the dimer PKM2 nuclear translocation was blocked.

### 3.5 β‐Elemene inhibited EGFR and GLUT1 signalling pathway

**Figure 6:**
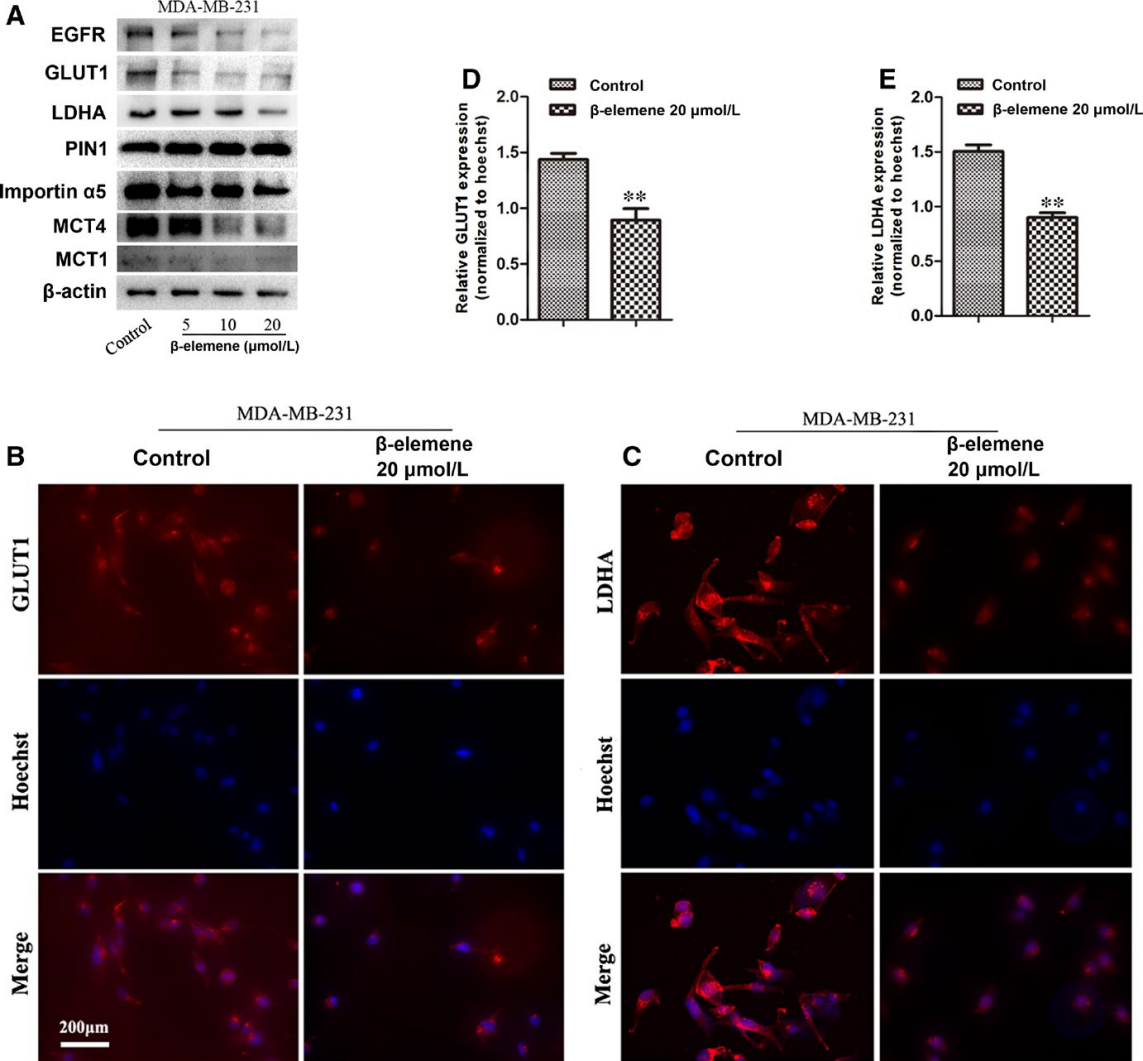
β‐Elemene inhibited EGFR and GLUT1 signalling pathway. A, MDA‐MB‐231 cells were treated with β‐elemene in indicated concentrations for 24 h, followed by Western blotting for EGFR, GLUT1, LDHA, PIN1, importin α5, MCT4 and MCT1 with indicated antibodies. B, C, Representative immunofluorescence staining (200×) of GLUT1 (B) and LDHA (C) in MDA‐MB‐231 cells treated with or without 20 μM β‐elemene for 24 h. D, E, The immunofluorescent intensity of GLUT1 (D) and LDHA (E) was quantitated by Mantra workstation with InForm Image software (PE corporation, USA) and analysed by GraphPad Prism. The quantitated data were for one‐way ANOVA with one side *t* test, versus control, **P < .01

Epidermal growth factor receptor (EGFR) activation has been considered to induce PKM2 translocating into the nucleus, leading to the up‐regulation of glucose transporter 1 (GLUT1) and lactate dehydrogenase A (LDHA) that jointly promote the aerobic glycolysis in a positive feedback loop.^29^ And this process is dependent on the extracellular signal–regulated kinase (ERK) signalling mediated by importin α5.^30^ Here, it was found that β‐elemene decreased the expression of EGFR, GLUT1 in a concentration‐dependent manner, as well as LDHA in both breast cancer cells (Figure 6A‐E, Figure S4A‐E). And β‐elemene also decreased expression of importin α5, but not its upstream PIN1 (Figure 6A, Figure S4A). The above‐mentioned data suggested the EGFR‐mediated signal pathway and GLUT1‐mediated glycolytic loop was broken. Additionally, the monocarboxylate transporter 1 (MCT1) and MCT4 responsible for the lactate balance of cells,^31^ but regulated by LDHA were inhibited by β‐elemene (Figure 6A, Figure S4A). The EGFR and GLUT1 signalling pathways regulating the aerobic glycolysis were blocked by β‐elemene.

### 3.6 Inhibitory effect of β‐elemene on breast cancer cells could be reversed by FBP and L‐cysteine

**Figure 7:**
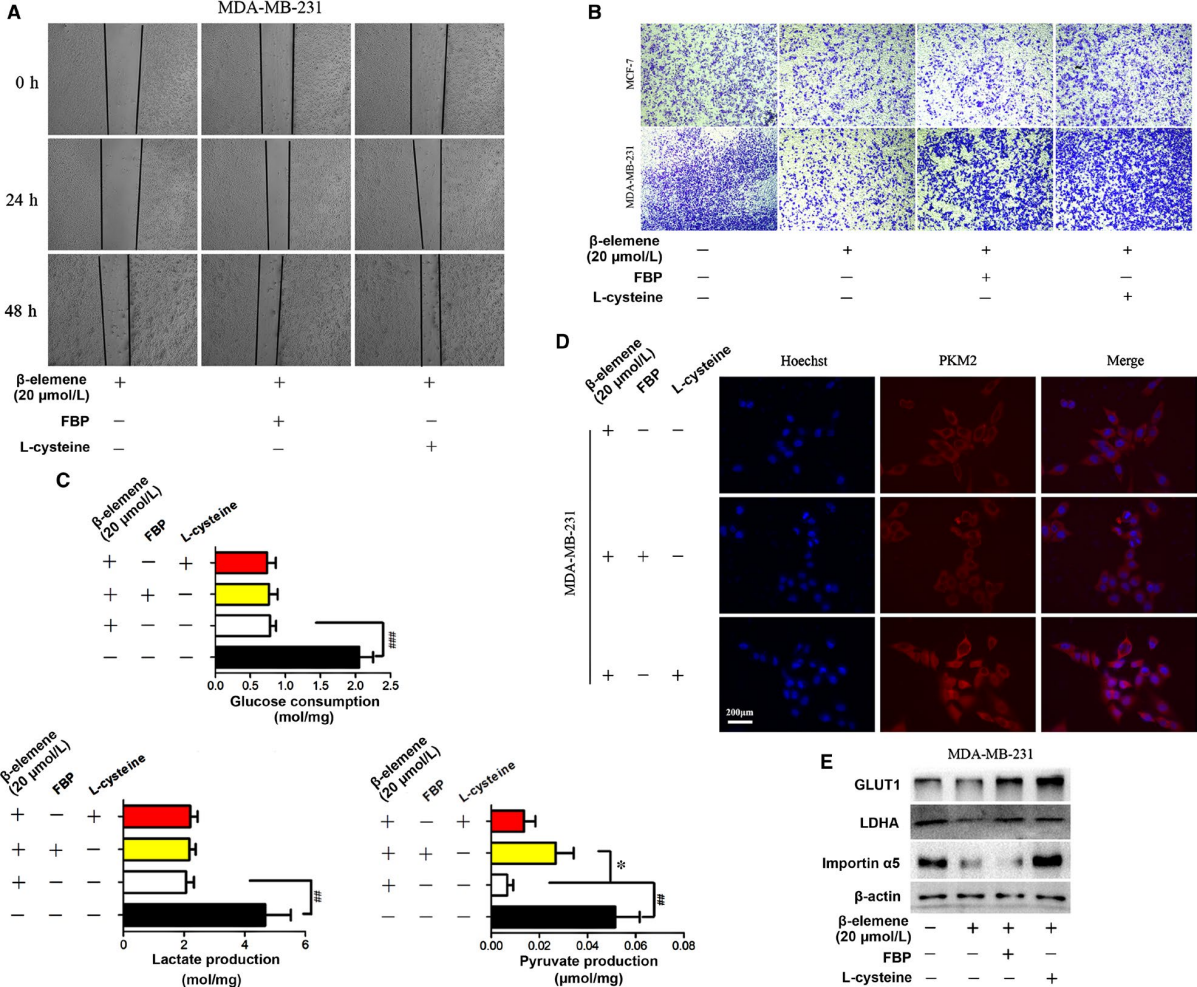
FBP and L‐cysteine reversed inhibitory effect of β‐elemene on breast cancer cells. MDA‐MB‐231 cells were treated with 20 μmol/L β‐elemene for 24 h in the presence or absence of FBP (100 μM) or L‐cysteine (100 μM), followed by analysis of the cell migration (A), invasion (B), metabolites (C), immunofluorescent staining of PKM2 (D), and by Western blotting for GLUT1, LDHA and importin α5 (E). The quantitated data were for one‐way ANOVA with one side *t* test, * *P* < .05, ^##^ *P* < .01

To further demonstrate the above results, the tetramer activator FBP and dimer activator L‐cysteine were used. Both activators could reverse the inhibitory effect of β‐elemene on migration and invasion (Figure 7A,B, Figure S5A,B). And the inhibition of β‐elemene on pyruvate production was only reversed by FBP (Figure 7C, Figure S5C), while that of β‐elemene on PKM2 nuclear translocation was blocked by L‐cysteine (Figure 7D, Figure S5D). These results further supported that β‐elemene inhibition of cell migration and invasion is mediated by reducing the ratio of tetrameric/dimeric PKM2 and the nuclear translocation of dimeric PKM2. Similarly, β‐elemene decrease of GLUT1 and importin α5 could be interrupted significantly by L‐cysteine in high metastatic MDA‐MB‐231 cells (Figure 7E), while FBP just reversed the GLUT1 expression (Figure 7E). However, in low metastatic MCF‐7 cells the significant reversal could not be observed (Figure S5E). They suggested that both EGFR and GLUT1 signalling pathway were also essential for the cancer cells metastases.

## 4 DISCUSSION

**Figure 8:**
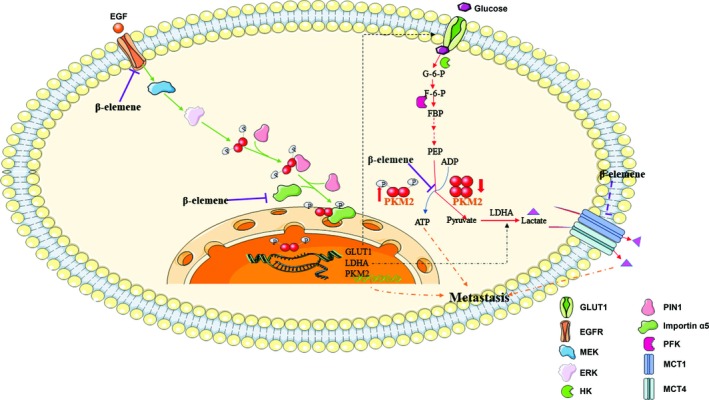
A diagram illustrating the molecular mechanisms of β‐elemene inhibition of breast cancer metastasis. β‐Elemene inhibited breast cancer metastasis by blocking aerobic glycolysis. Mechanistically, β‐elemene prevented the transformation of dimeric and tetrameric forms of PKM2, thus inhibiting its pyruvate kinase activity, leading to decreased utilization of glucose and production of pyruvate/lactate. Also, β‐elemene suppressed EGFR‐importin α5‐mediated nuclear translocation of PKM2 and expression of GLUT1, MCT1, MCT4 and LDHA

It was reported that PKM2 can promote cell migration through activation of PI3K/AKT pathway in gastric cancer cells,^32^ and deregulation of PKM2 also enhances the metastatic activity in tongue squamous cell carcinoma,^33^ indicating that PKM2 is an important factor to regulate cancer metastasis. In this study, we found that the ATP production was significantly different between high and low metastatic breast cancer cells, proving that the metastasis is related to energy metabolism. One of the mechanisms that monitor the glycolytic phenotype is the tight regulation of the enzyme PKM2.^8^ PKM2 transforms between a tetramer with high activity and a dimer with low activity, the former having high affinity to PEP but the latter having low affinity to PEP.^9^ In cancer cells, the ratio of tetramer/dimer of PKM2 controls the metabolic pathway of glucose which is accessed for producing energy or for synthesizing anabolic precursors regulated by metabolic intermediates, such as fructose 1, 6‐biphosphate, serine, SAICAR and post‐translational modifications, including phosphorylation, acetylation and oxidation.^34^, ^35^ And the interconversion between dimeric and tetrameric PKM2 keeps dynamic balance. Here, we found that β‐elemene affected the conversion of two forms of PKM2 to block the glycolysis (Figure 8). Apart from its metabolic role, PKM2 also functions as a non‐metabolic protein kinase and transcriptional coactivator for c‐MYC and HIF‐1α which are essential for EGFR activation‐induced tumorigenesis. Activated EGFR promotes PKM2 nuclear translocation dependent on importin α5.^30^ In our research, β‐elemene inhibited the activity of PK and the nuclear translocation of PKM2 by down‐regulating importin α5. Furthermore, the expression of EGFR was also decreased, supporting that the EGFR‐importin α5 signalling pathway is involved in β‐elemene inhibition of breast cancer metastasis (Figure 8).

Currently, two therapeutic strategies targeting PKM2 are developed in preclinical research: one is to look for the inhibitors of PKM2 catalytic activity, and another is for the activators of tetramerization of PKM2 to enhance glycolysis.^7^ However, both have no entrance into human clinical studies till now. β‐Elemene is less cytotoxic and could enhance the sensitivity to radiotherapy and chemotherapy.^36^, ^37^ Although the inhibition of PKM2 could lead to inhibition of cancer cell proliferation, most of cancer cells could survive by another compensatory glutaminolysis way.^38^ Whether β‐elemene can overcome this problem needs further examination. Clearly, it would have a great impact on clinic practice to develop combinational therapeutic regimens that can target the several metabolism pathways that are essential for maintaining the malignant state of breast cancer.

Lactic acid is the product of anaerobic glycolysis. However, many physiological cells, including erythrocytes, lymphocytes, white muscle fibres and pathologic cells, such as cancer cells, rely on glycolysis providing their energy even in the presence of oxygen.^39^ It has been reported that oxygenated cancer cells expressing MCT1 import lactate and oxidize it to produce energy.^40^ Lactate not glucose was preferentially overconsumed for fuelling TCA cycle in human non‐small‐cell lung cancer expressing MCT1 and supporting the cancer metabolism in vivo,^41^ indicating that lactate can serve as a potential nutrient source for tissues and tumours. 18 ATP yielded by a molecule lactate could meet the needs of glycolytic enzymes more concisely and effectively than glucose oxidation under aerobic condition.^40^, ^42^ However, this process could be interrupted by MCT1 inhibition. Inhibition of MCT1 resulted in oxidative cancer cells switching to glycolysis from lactate oxidation.^40^ Furthermore, the lactate and glutamate secreted by cancer cells are utilized and metabolized by cancer‐associated fibroblasts (CAFs) to synthesize lighter glutamine isotopologues for glutaminolysis and nucleotide synthesis in cancer cells.^43^ The symbiotic relationship between oxidative, glycolytic tumour cells and CAFs is formed by lactate. In our study, β‐elemene selectively inhibited MCT1 and MCT4 in different metastases (Figure 8), which may disrupt the symbiotic relationship, making tumour cells difficult to survive in the metastases.

Although inhibition of PKM2‐mediated glycolytic pathway links to the anti‐metastatic effect of β‐elemene, it was still hard to exclude the possibility that other metabolic pathways (eg the pentose phosphate pathway) are also involved in this event. Glycolytic intermediates including G6P can enter the pentose phosphate pathway (PPP), which participates in the synthesis of macromolecules such as nucleotides. Besides, minority of fructose‐6‐phosphate (F6P) could be converted to the PPP by glucose‐6‐phosphate isomerase.^44^, ^45^ In our research, β‐elemene decreased both ECAR and OCR in MCF‐7 cells, but the cancer cells still survived. G6P was decreased by β‐elemene in MCF‐7 cells. However, F6P and 3‐phosphoglycerate (3PG) were not influenced, suggesting that β‐elemene might activate PPP. Thus, likely, activation of PPP may also be a possible way to inhibit breast cancer metastasis.

Additionally, pharmacokinetics studies have shown that the lung, liver and brain are the top three tissues of β‐elemene distribution.^46^ This suggested that β‐elemene may be clinically applicable to patients with primary carcinoma in lung/liver as well as those with breast cancer metastases in lung/liver. Collectively, our results indicated that β‐elemene inhibits metastasis of breast cancer by blocking PKM2‐mediated metabolic signalling, being a potential anti‐metastatic agent.

## CONFLICTS OF INTEREST

The authors declare that they have no conflict of interest.

## AUTHORS' CONTRIBUTIONS

WXC and YL designed the study; WXC wrote the manuscript. YHP, WW, SH, WTN, ZHW, YZC, QJ, YYW conducted majority of experiments. SYY conducted the data enrichment analysis. CC completed the HPLC analysis. SJW analysed the pathological data. QZ and LZ prepared the figures and performed the statistical analysis. AYW participated co‐ordination. SLH and ZGS participated in its design and co‐ordination. All authors read and approved the final manuscript.
