# Supplementary figures and images for: Beta‐elemene inhibits breast cancer metastasis through blocking pyruvate kinase M2 dimerization and nuclear translocation
Source: J Cell Mol Med. 2019 Jul 25;23(10):6846–58. doi: 10.1111/jcmm.14568 (PMC6787513; doi:10.1111/jcmm.14568)

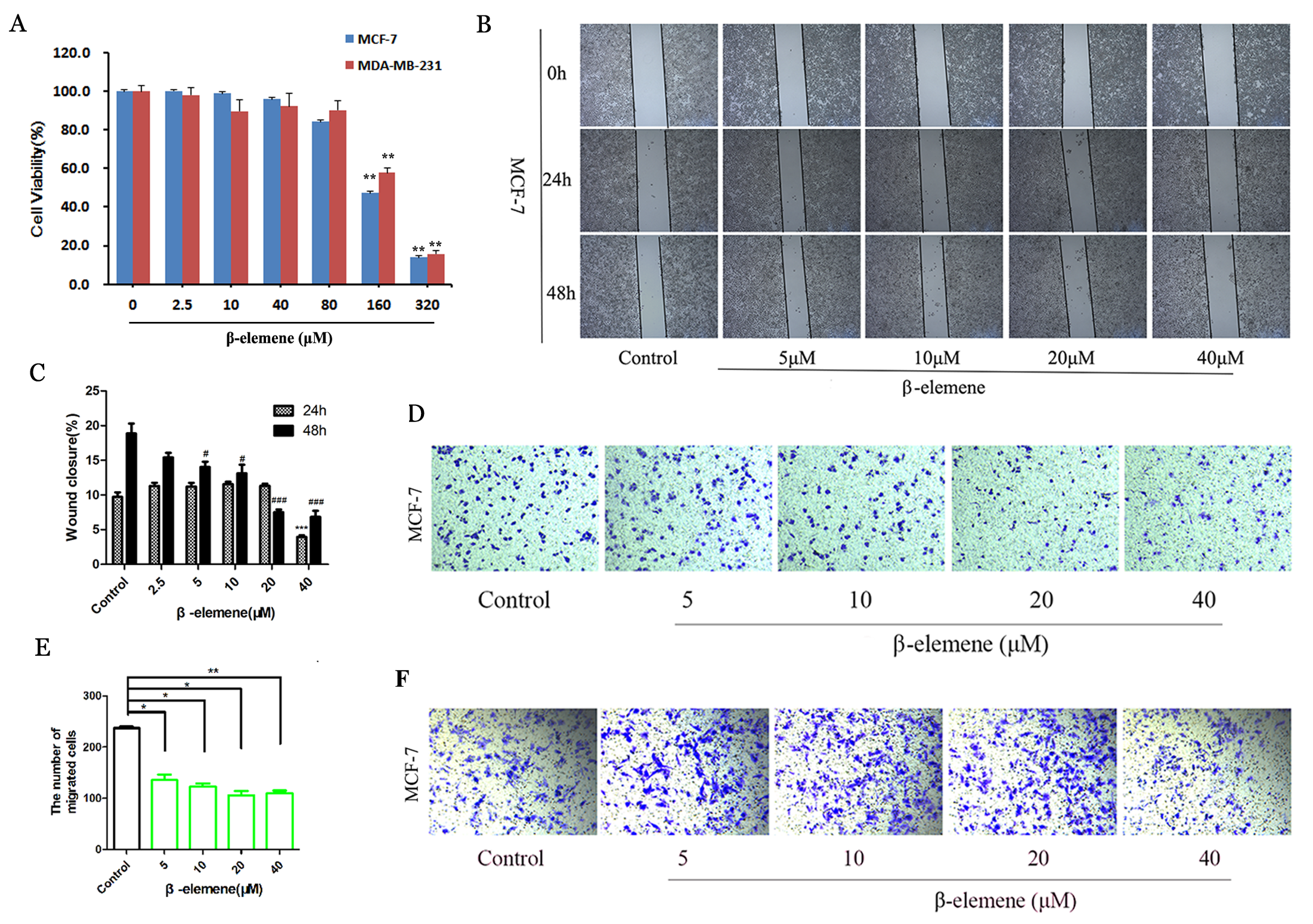

Supplement: Supplementary file 1 [file JCMM-23-6846-s001.tif]

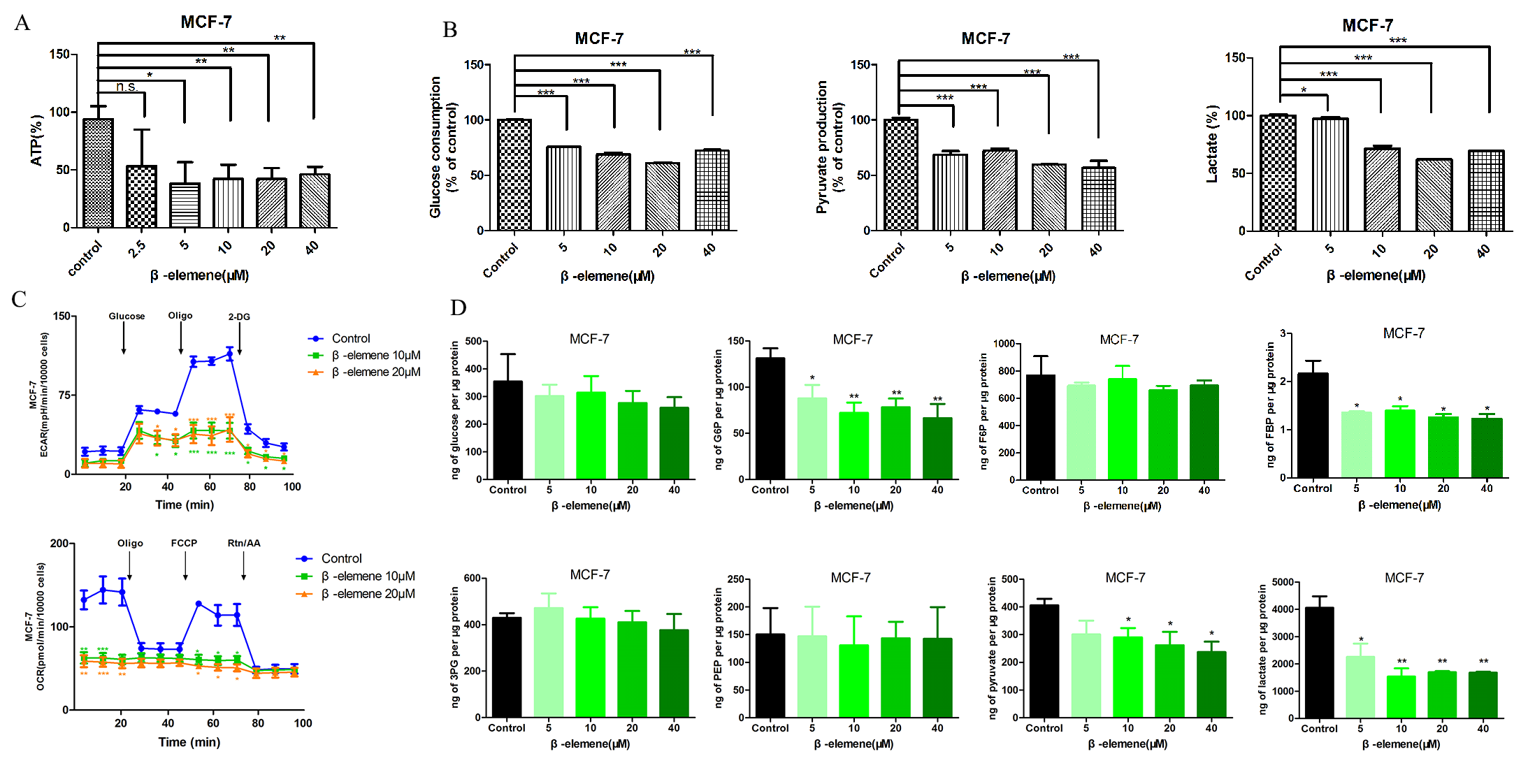

Supplement: Supplementary file 2 [file JCMM-23-6846-s002.tif]

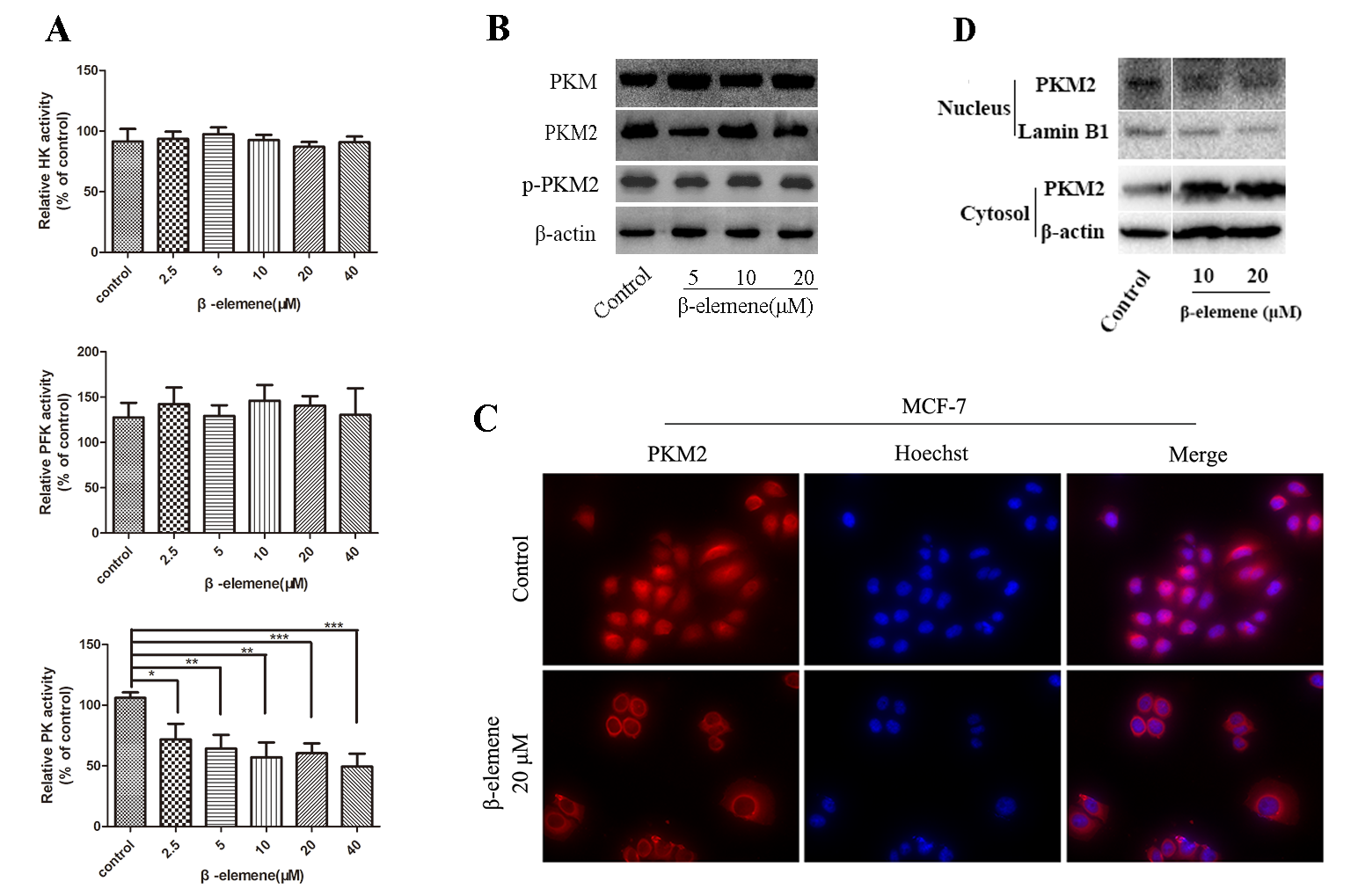

Supplement: Supplementary file 3 [file JCMM-23-6846-s003.tif]

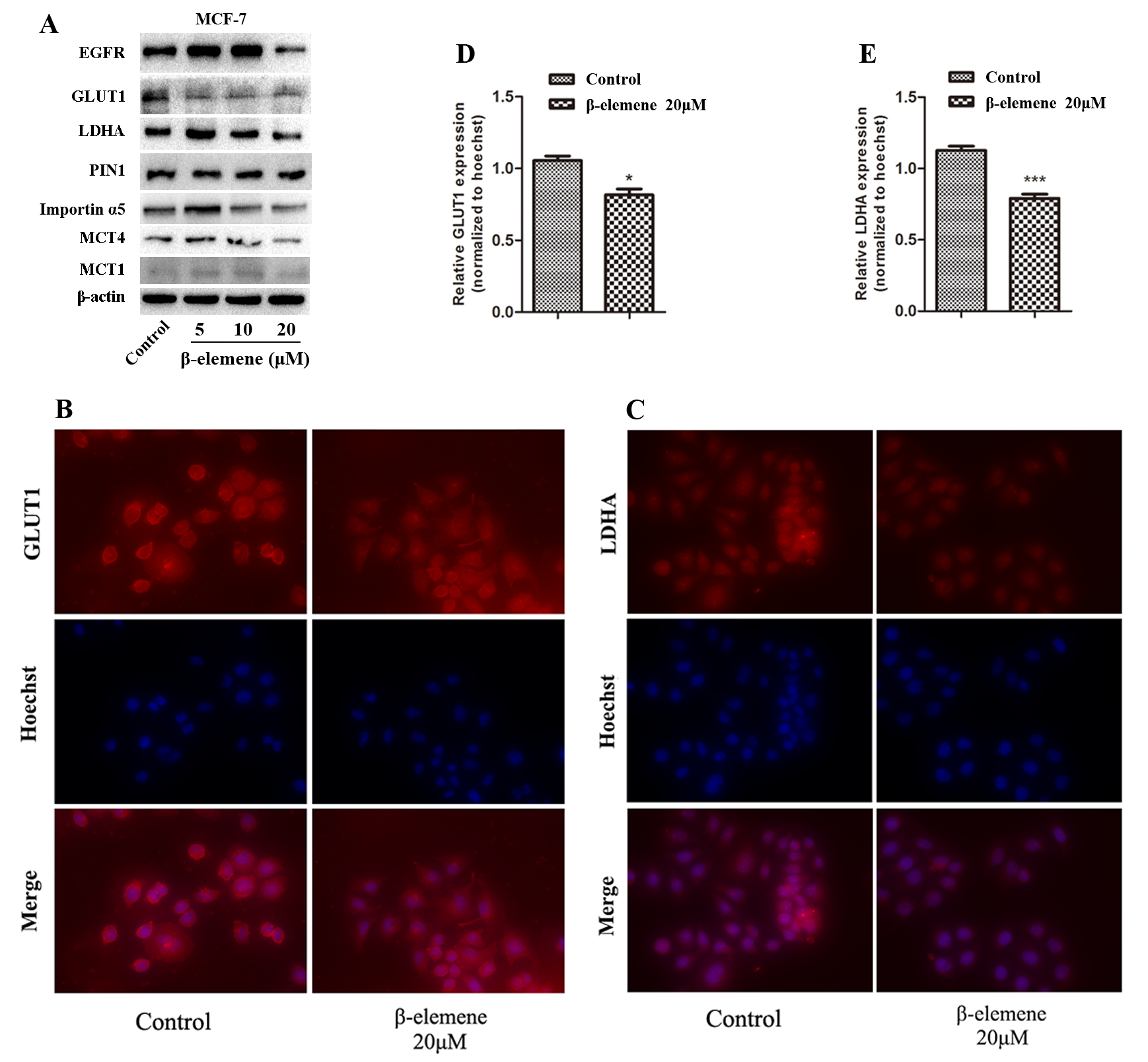

Supplement: Supplementary file 4 [file JCMM-23-6846-s004.tif]

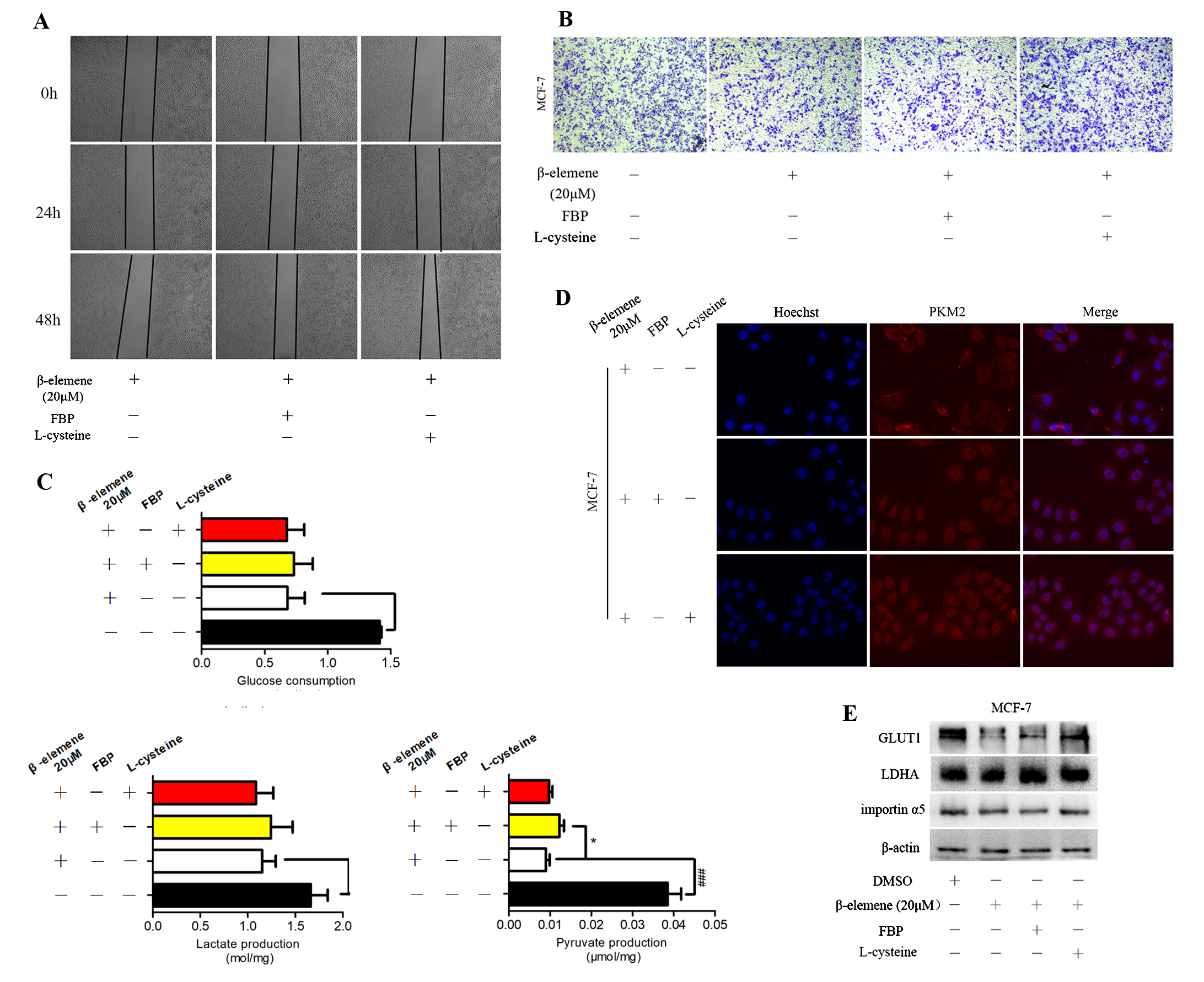

Supplement: Supplementary file 5 [file JCMM-23-6846-s005.tif]
